# Validation of Growth Layer Group (GLG) depositional rate using daily incremental growth lines in the dentin of beluga (*Delphinapterus leucas* (Pallas, 1776)) teeth

**DOI:** 10.1371/journal.pone.0190498

**Published:** 2018-01-16

**Authors:** David A. Waugh, Robert S. Suydam, Joseph D. Ortiz, J. G. M. Thewissen

**Affiliations:** 1 Department of Anatomy and Neurobiology, Northeast Ohio Medical University, Rootstown, Ohio, United States of America; 2 North Slope Borough, Department of Wildlife Management, Barrow, Alaska, United States of America; 3 Department of Geology, Kent State University, Kent, Ohio, United States of America; Augusta University, UNITED STATES

## Abstract

Counts of Growth Layer Groups (GLGs) in the dentin of marine mammal teeth are widely used as indicators of age. In most marine mammals, observations document that GLGs are deposited yearly, but in beluga whales, some studies have supported the view that two GLGs are deposited each year. Our understanding of beluga life-history differs substantially depending on assumptions regarding the timing of GLG deposition; therefore, resolving this issue has important considerations for population assessments. In this study, we used incremental lines that represent daily pulses of dentin mineralization to test the hypothesis that GLGs in beluga dentin are deposited on a yearly basis. Our estimate of the number of daily growth lines within one GLG is remarkably close to 365 days within error, supporting the hypothesis that GLGs are deposited annually in beluga. We show that measurement of daily growth increments can be used to validate the time represented by GLGs in beluga. Furthermore, we believe this methodology may have broader applications to age estimation in other taxa.

## Introduction

**Fig 1:**
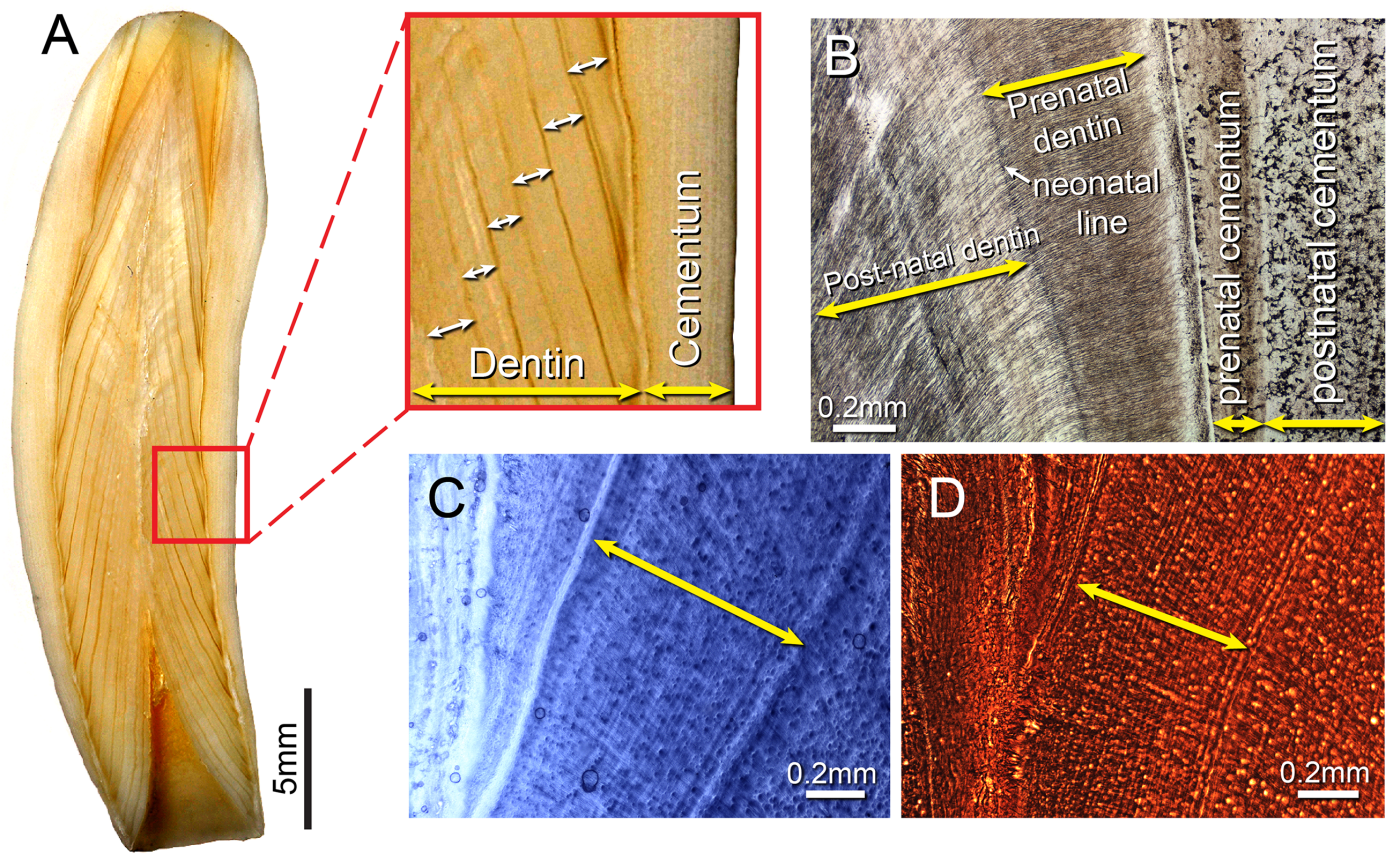
Beluga tooth basic histology and Growth Layer Groups. (A), half tooth (2014LDL8T4), cut longitudinally and polished, GLGs marked with white double-headed arrows in magnified view delimited by red box; (B), ground section (2014LDL8T3) with histological features labeled; (C-D), demineralized sections (2012LDL8T2), GLGs marked with yellow double-headed arrows; (C), stained with Mayer’s hematoxylin; (D), and Bielschowsky’s silver stain.

Counts of growth layers in the dentin of marine mammal teeth are widely used as indicators of age [1,2]. The most prominent growth layers in cetacean teeth occur in the dentin (Fig 1A), and in most cetacean species these layers form annually [1,3–5]. However, with respect to beluga whales, the periodicity of deposition of these large-scale growth layers has remained the subject of uncertainty. In this study, we used short-period incremental lines [6] that are assumed to represent daily pulses of dentin mineralization to test the hypothesis that large-scale growth layers in beluga dentin are deposited on a yearly basis.

The use of dentin growth layers to assess the age of marine mammals began in the 1950s and was rapidly adopted as a valid age assessment tool in toothed-whale populations [2,7]. In an effort to standardize terminology used in age estimation of marine mammals, Perrin and Myrick [8] labeled the large-scale repeating patterns in the dentin as: *Growth Layer Groups* (GLGs). The term Growth Layer Group, by definition, does not specify a unit of time in which the dentin was deposited [8]. However, in most marine mammals one GLG has been shown to encompass the hard tissue deposited during one year [1,3–5]. Unlike the teeth of most mammals, beluga teeth, as well as those of sperm whales, possess an open pulp cavity that allows dentin to be deposited through most of the animal’s life [9] producing an unusually long record of near continuous dentin deposition. Thus, an accurate count of the GLG’s contained in a tooth provides an age estimate in years if a complete record of dentin is preserved.

The ability to accurately estimate the age of an animal is vital for the assessment and management of wildlife populations [10–12] and is of special importance in assessing and managing populations that are threatened or live in fragile or changing environments like the Arctic [13,14]. In addition, other intrinsic and extrinsic events or signals may be recorded within dentin [3,15]. In cetaceans, information shown to have been recorded in dentin include: birth [15–17]; sexual maturity [18–21], weaning [20,21] lactation [22], and environmental events [23–25]. Thus understanding the temporal framework of growth layers in dentin is the first step in placing these other signals in a usable context.

Sergeant (1959) studied beluga teeth and tentatively proposed that belugas deposit two semi-annual GLGs per year; this view is supported by some studies [9,26–31]. However, other studies support the view that a single GLG is formed each year [4,5,12,32–36].

In prior studies to validate the periodicity of cetacean GLGs, the teeth from either known-age individuals, or teeth from individuals administered tracers such as the antibiotic tetracycline at specific intervals, typically from captive populations, were examined [15,19,37–41]. In beluga, tetracycline-based studies have either been too short, or wild animals administered tetracycline could not be recaptured. Beluga in captivity may produce teeth with poorly formed GLGs (i.e. [4,26,29]) and the poor readability of these teeth has hampered studies of both known-age animals and those administered tetracycline. In some cases, only the beluga’s time in captivity is known, because the age at time of capture is unknown, or loosely constrained. In addition, if the beluga’s tooth wear has progressed to the point of eroding the neonatal line (Fig 1B) or subsequent GLGs, resultant counts provide only a minimum age, [4,9,31,42] complicating studies involving known-age animals. Thus, methods used successfully to established the time represented by GLGs in other cetaceans have not produced universally accepted results concerning age assessment in beluga, mostly due to a lack of suitable specimens. Newer methods of age estimation such as aspartic acid racemization [43] and analysis of fatty acids [44] are under development for use with beluga, but they still require calibration with known-age animals or must rely on some other independent method of age assessment.

Other methodologies used to determine duration of GLG formation in beluga have involved comparing growth curves and life-history events such as age of sexual maturity to those of other cetaceans [12,34], the use of growth layers in the mandible [31], and comparing histories of isotopic signals in dentin with other species of known-age [35]. Stewart et al. [33] used the appearance of a ^14^C spike produced by the atmospheric testing of atomic bombs in the 1960’s and its incorporation into beluga dentin as a time marker and found support for the one GLG per year hypothesis

Short-period incremental lines have been experimentally shown to represent daily deposits during periods when teeth are forming in humans [45], other primates [46–48], pigs [49], dogs [47,48], alligators [50], rats [47,48,51], rabbits [52], mice [47,48], and voles [53]. These daily growth lines have also been referred to as “von Ebner lines”. Because this term has been used by various authors to describe lines of different physical scales and periodicities [6,54], for the sake of clarity, we avoided using it; other than to discuss its usage in the literature as related to cetaceans.

Few studies have reported the presence of short-period growth lines in the dentin of cetaceans. Keil and von Nolting [55] describe ‘incremental lines’ in whale dentin although they do not address the periodicity, their description suggests that they are describing the same type of incremental lines that we observed and illustrated in beluga (see Discussion). In the dolphin *Stenella attenuata*, Myrick [56] reported the presence of ‘von Ebner’ lines which he interprets as having a daily periodicity. His study is the first, to our knowledge, to recognize these short-period incremental growth lines as daily growth lines in cetacean dentin. Myrick [56] found that each GLG was subdivided by intermediate-scale layers into 13 sub-units, which he attributed to lunar cycles. Within the 13 sublayers, he counted between 27–28 von Ebner lines, which support his interpretation that they represent daily growth increments, an interpretation with which Hohn [7] agreed, and we concur. Klevezal (1996, p.9 fig.5) shows a ground beluga tooth section with a caption indicating the presence of ‘von Ebner’ lines. In the text she suggests these layers are analogous to the intermediate-scale ‘lunar’ sub-units of Myrick [56]. From the context, it is clear that Klevezal [3] is suggesting the lines she figures are intermediate in scale and do not correspond to the daily lines described by Myrick. This seeming contradiction illustrates the divergent usage of the term “von Ebner lines”, and explains why we refer to the lines described in this study simply as short-period, or daily, incremental lines.

To test the emerging consensus that beluga whales form one GLG per year, we used short-period incremental lines [6] preserved in the dentin to estimate the time represented in one GLG. By counting the number of short-period lines relative to GLG’s we can establish the timescale of both the GLG as annual, and the short-period incremental lines as daily, a methodology that to our knowledge has not been applied in marine mammal teeth.

Of the growth lines we observed in our material, the finest (short-period incremental lines) and the coarsest (GLGs) are sufficiently well-defined in our samples to be quantified at this time. We thus focused on analysis of the daily incremental growth lines and GLGs observed in the samples for this study.

We report the presence of short-period incremental growth lines, which we interpret as daily in beluga dentin, and have used them to estimate the number of days represented by one GLG. If one GLG represents one year’s growth, the expected result would be 365 daily growth increments in one GLG. Conversely, if two GLGs are formed in one year, the placement is semi-annual, and half the number of increments are predicted within a single GLG. In addition to using daily growth lines to successfully test a specific hypothesis in beluga, we suggest that the methodology may have broader applications to age assessment in other taxa, and in the observation of sub-yearly signals that may be recorded in dentin.

## Materials and methods

### Material collection

Beluga from the eastern Chukchi Sea population [57,58] were sampled by the North Slope Borough Department of Wildlife Management (NSB-DWM) and its collaborators, under NOAA-NMFS permit (17350–00 to NSB-DWM). Iñupiat subsistence hunters of Point Lay, Alaska, harvest belugas on their migration North from the Bering to the Chukchi and Beaufort seas in late June or early July [59]. The NSB-DWM collects samples, including teeth, and records biometric data from these whales to assess population health. Teeth were extracted by boiling or macerating the mandible and teeth were stored, unfixed, either dry or in water. One tooth from each of three whales that had been previously sectioned for age estimation were selected for decalcification and histologic staining (Table 1). We numbered (ex. 2012LDL8T4) individual teeth with the year of collection, “LDL” (Point Lay, *D**elphinapterus* *l**eucas*), the sequential whale number, and a “T” (for tooth) followed by the mandibular tooth number. Teeth are numbered starting from the most anterior part of the mandible [60].

**Table 1:** Specimen information and tabulated measurements.

Sample data				GLG	Incremental line measurements			
Specimen	Sex	Tooth pos.	Whale length (cm)	Thickness (μm)	Average (μm)	3σ_SE_	Sum (μm)	Incr. (n)
2012LDL3-a	F	6	362	482.00	1.47	0.08	178.30	121
2012LDL3-b	F	6	362	408.82	1.41	0.09	170.58	121
2012LDL8-a	M	2	385	700.30	1.79	0.15	201.95	113
2012LDL8-b	M	2	385	711.62	2.05	0.14	214.73	105
2012LDL8-c	M	2	385	707.62	1.87	0.12	411.55	220
2009LDL10-a	M	3	334	531.00	1.46	0.08	203.32	139
2009LDL10-b	M	3	334	573.63	1.50	0.10	154.93	103
**Average**			**363.86**	**587.86**	**1.65**	**0.11**	**219.34**	**131.71**

Specimen number is followed by a letter indicating the specific GLG measured (each measured GLG is unique, even if from the same tooth). 3σ_SE_ = 3 times the standard error for the incremental line average; Sum, is the cumulative thickness of the incremental lines; Incr., Indicates the number of daily incremental lines measured within each GLG.

### Ground sections

Teeth were cut longitudinally using a water-cooled diamond saw. One surface was polished using successively finer abrasive disks (diamond abrasives bound in resin), and then finished with silicon carbide abrasive paper (P4000 grit) on a water-cooled lap. Polished faces were thoroughly dried and glued to frosted slides with a UV curing resin (Loktite 349). Remaining tissue was sawn off the slide, leaving around 1 mm of material, which was subsequently ground and polished to a final thickness of between 30–50 μm using successively finer abrasives as described above.

### Demineralized sections

Teeth, which had previously been sectioned longitudinally close to the central axis, were fixed in 10% neutral buffered formalin for a period of a least 5 days, rinsed in deionized water, and demineralized in a buffered formic acid solution [61]; decalcification endpoints were determined by x-ray inspection. Teeth were then rinsed to remove the formic acid and placed overnight in a 30% sucrose solution made with phosphate buffered saline as a cryoprotectant, before being frozen onto the dry-ice cooled stage of a sliding microtome (American Optical 860), and serially sectioned at 20 μm. Sections were removed from the knife with a soft brush and placed in phosphate buffer. After sectioning, slices were stored in 70% ethyl alcohol at 4°C until staining.

In sections stained with Mayer’s hematoxylin (described below), those that were too thin did not take up enough stain to provide the needed contrast, while sections that were too thick became opaque enough to hinder measurement. Sections of 20 μm provided the clearest contrast for sections stained with either silver or hematoxylin.

#### Mayer’s hematoxylin

Sections were rinsed in water and stained free-floating in Mayer’s Hematoxylin [62] in small vials for between 12–48 hours, and rinsed in water. Sections were then blued in Scott’s Tap Water Substitute [63], rinsed, and floated onto chrome alum gelatin subbed slides. Mounted sections were air-dried overnight, briefly cleared in xylene, and cover-slipped with Permount (Fisher) or DPX as a mountant. We followed Myrick et al. [64] and Evans et al. [65] and excluded the chloral hydrate and citric acid from the Mayer’s hematoxylin formulation; but, we did use the full amount of sodium iodate called for in Mayer [62]. The recipe given by Myrick et al. [64] contains half the amount of sodium iodate as used by Mayer [62] and Evans et al. [65]. The long staining times did not result in overstaining of the section as a whole, but did increase the stain taken up by the incremental lines. No differentiation step with acid alcohol was needed. With the longer staining times employed, attempts to stain sections already attached to subbed slides produced uneven results.

#### Bielschowsky’s silver stain

Although intended for the staining of neural fibers, particularly in brain tissue, silver stains have been found to stain incremental lines in dentin [45,66,67]. Bielschowsky’s techniques can be applied to whole blocks of tissue (before sectioning), or to individual sections, both methods have been used to examine incremental lines in teeth. Because we wanted to use multiple stains for different slices of the same specimen, we chose to stain sections individually.

Loose, unstained sections were rinsed in water, floated onto chrome alum gelatin subbed slides, and air-dried for at least 12 hours before staining with a Bielschowsky silver stain modified by Mirra et al. [68] used for the diagnosis of Alzheimer’s disease [68]. We modified the time that sections spent in different reagent baths to account for the thicker sections and different tissue. We increased the time in the 20% silver nitrate to 20 from 15 minutes, and increased the time in the 5% sodium thiosulfate from 2 to 5 minutes. After the final rinse, the slides were air-dried overnight, cleared with xylene and cover slipped with Permount.

Silver stains have a reputation for being difficult to reproduce [69]. Our experience reinforced this observation and our (DAW) first attempts at using the silver stain produced poor results. Upon the recommendation of René Buesa (personal communication) we added a few drops of (reserved) silver nitrate solution back into the ammoniacal silver solution after the precipitate from the sodium hydroxide had just been dissolved, insuring the correct amount of silver and keeping the ammonia levels in check, which might otherwise detach sections from the slide. This step seemed to increase the repeatability of the protocol. It was found that after demineralization, a secondary fixation step in 10% neutral buffered formalin was needed, either before or after sectioning, for the silver to adequately stain the tissue. At this time, it is unclear if longer initial fixation times would eliminate the need for a second fixation step after demineralization.

### Microscopy and image analysis

Photomicrographs for quantification of incremental line spacing were taken on a Zeiss Imager M2 compound microscope equipped with a high-resolution CMOS camera (monochrome) and motorized stage. Images of the daily short-period lines were taken with a 63X oil immersion objective as virtual stacks of around 20 images with a z-spacing of 0.2–0.3 μm, producing a stack of images with successive deeper focal planes. Short-period lines are more distinct in some focal planes than others, hence the z-stack improves our resolving abilities over having to pick a single plane of focus. Images of the Mayer’s hematoxylin stained sections were taken with a green filter in the light path to improve image contrast.

The one ground section was imaged under differential interference contrast on a compound microscope (Olympus BX60) and camera (monochrome), using a 40X oil lens. A coverslip was temporarily attached using glycerin and regular immersion oil was used between the coverslip and the objective. The lower index of refraction of the glycerin improved the contrast over use of immersion oil between the cover glass and the tissue.

The computer software Fiji [70], a variant of NIH ImageJ, was used for image analysis and measurement. The Pairwise Stitching plugin [71] written for Fiji was used to “stitch” adjacent image stacks together producing one stack covering the entire width of one GLG (typically 4–5 stacks). Measurements of incremental line spacing were made in Fiji. As different focal planes offered the best clarity for a given region it was impossible to measure all the lines in a single focal plane. Images figured in this paper were prepared using the 3D EDF plugin [72] for Fiji. This plugin takes the in-focus areas from an image stack and merges them together to produces a composite image containing only the focused regions.

**Fig 2:**
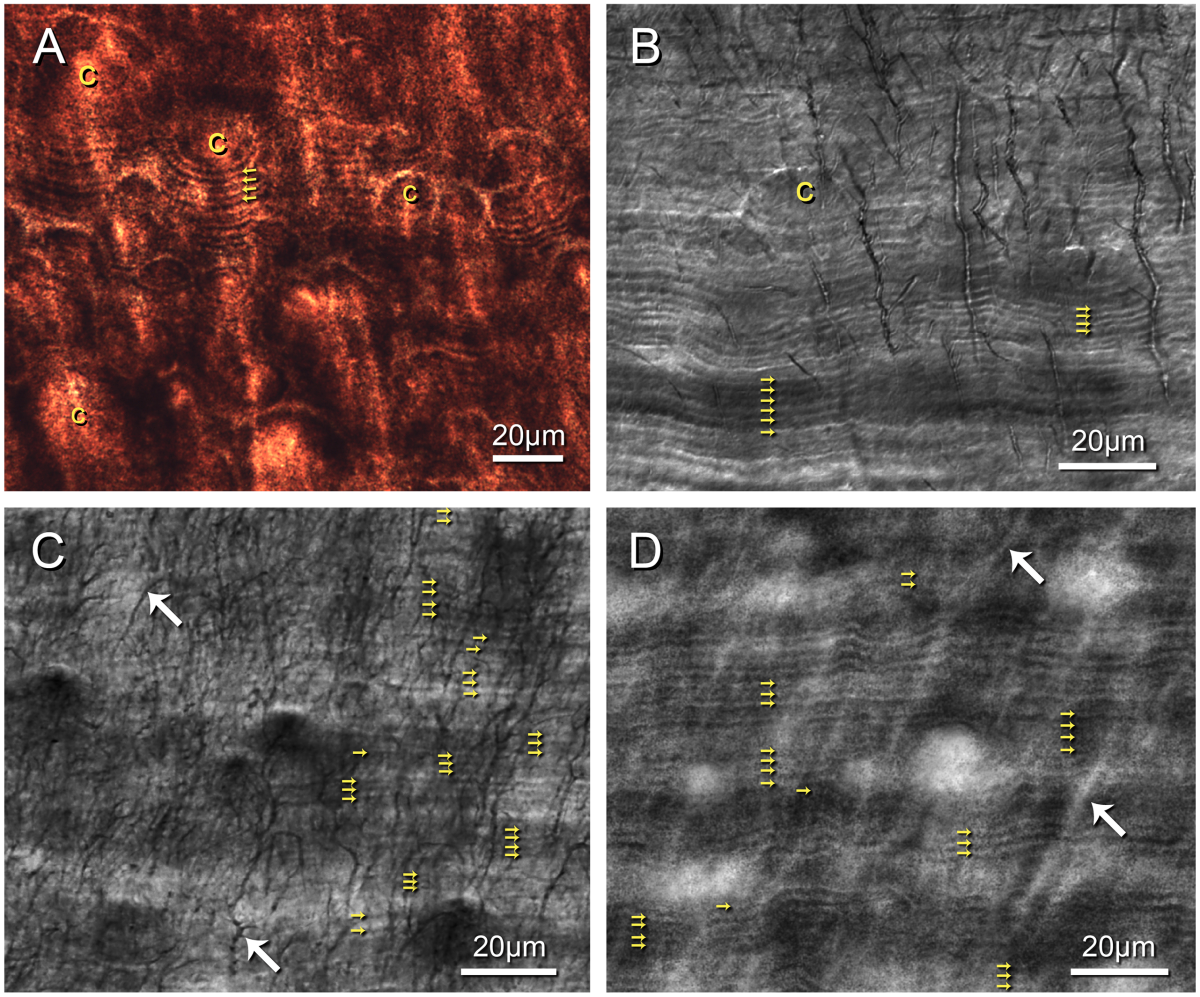
Daily incremental lines. (A-D), daily incremental lines (yellow arrows) in beluga dentin. (A), incremental lines following the contours of calcospheres (c), demineralized section (2012LDL8T2), Bielschowsky’s silver stain, monotone photomicrograph; (B), ground section (2014LDL8T3), differential interference contrast, monotone photomicrograph; (C), Incremental lines and dentin tubules (white arrows) demineralized section (2012LDL3T6), Mayer’s hematoxylin, monotone photomicrograph; (D), Incremental lines demineralized section (2012LDL8T2), Bielschowsky’s silver stain, monotone photomicrograph.

Seven unique GLGs from three teeth were selected for the quantification of incremental line spacing based on the clarity of the incremental lines within a given GLG, and the certainty in which the boundaries of the GLG could be identified. GLGs that had ambiguous boundaries were avoided. Some incremental lines had indistinct boundaries, and we chose to measure the distance between two lines from the center of one dark line to the next, as the center of each line could be picked more precisely in the photomicrographs. Only one measurement was taken between any two incremental lines. Total GLG thickness was measured in the same image stack as the incremental line spacing. All measurements were taken following the direction of the tubules (Fig 2C and 2D) which follow the direction of dentin growth [73–75].

We tested the hypothesis that one GLG is deposited annually by predicting the spacing between adjacent incremental lines under two scenarios, one in which a GLG represents one year, and one in which two GLGs are deposited on a semi-annual basis (Table 2). The predicted mean incremental line thickness for one GLG per year is simply total GLG thickness divided by 365. The predicted spacing between daily incremental lines if two GLGs are deposited per year is GLG thickness divided by 182.5 (Table 2). The estimated number of days represented by each GLG in our sample was calculated as the thickness of the GLG divided by the mean spacing of daily incremental lines within that GLG (Table 2). Errors are given as three times the standard error to provide a more conservative estimate of error in estimation of the average incremental line thickness and the estimated days per GLG and are justified by our relatively small sample size.

**Table 2:** Estimated and predicted values.

GLG	Average incremental line thickness (μm)	Predicted incremental line thickness (μm)		Estimated number of days in each GLG		
		1GLG/yr	2GLG/yr	Estimated days in GLG	3σ_SE_ (days)	
2012LDL3-a	1.47	1.32	2.64	327.10	-17.37	+19.44
2012LDL3-b	1.41	1.12	2.24	290.00	-17.32	+19.66
2012LDL8-a	1.79	1.92	3.84	391.84	-30.54	+36.18
2012LDL8-b	2.05	1.95	3.90	347.97	-21.81	+24.93
2012LDL8-c	1.87	1.94	3.88	378.27	-22.31	+25.29
2009LDL10-a	1.46	1.45	2.91	363.03	-18.89	+21.09
2009LDL10-b	1.50	1.57	3.14	381.36	-24.27	+27.81
**Average**	**1.65**	**1.61**	**3.22**	**354.22**	**-21.79**	**+24.91**

1GLG/yr represents the predicted distance between incremental lines if a GLG forms in one year; 2GLG/yr represents the predicted spacing if two GLGs form in one year; 3σ_SE_ = 3 times the standard error.

## Results

### Short-period incremental lines

In beluga, short-period incremental lines appear as alternating stained (dark) and unstained (light) layers in demineralized sections stained with either Bielschowsky’s silver or Mayer’s hematoxylin (Fig 2C and 2D). Although both methods stained the incremental lines, the Bielschowsky’s silver stain resulted in sections of slightly improved clarity (Fig 2D). For comparative purposes one undemineralized ground section was also examined (Fig 2B) and appeared to exhibit the same incremental lines as observed in the demineralized sections.

Most GLGs contained a high-density of readable incremental lines, while in others the expression and fidelity of these lines were variable, such that some GLGs exhibited almost a complete absence of readable incremental lines. Within approximately one third of the sections, individual lines could be followed along a single GLG for most of the section, in other regions the lines could vary from being sharply expressed, to indistinct, moving laterally a few tens of microns (Fig 2C and 2D).

In most sections the dentin contains regions of globular and inter-globular dentin (Fig 2, especially Fig 2A) expressed as incompletely fused calcospheres [76]. In regions containing calcospheres, incremental lines follow an arcuate path along the contours of the incomplete spheres (Fig 2). In all preparations, including the mineralized ground section, the increment lines showed the same relationship with the calcospheres. Mean spacing between incremental lines in the demineralized sections was 1.65 μm (3σ_SE_: 0.11) (taken from tabulated means in Table 1) and for the one non-demineralized ground section measured 2.31 μm (3σ_SE_: 0.36; n: 13).

### Validation of GLGs

**Fig 3:**
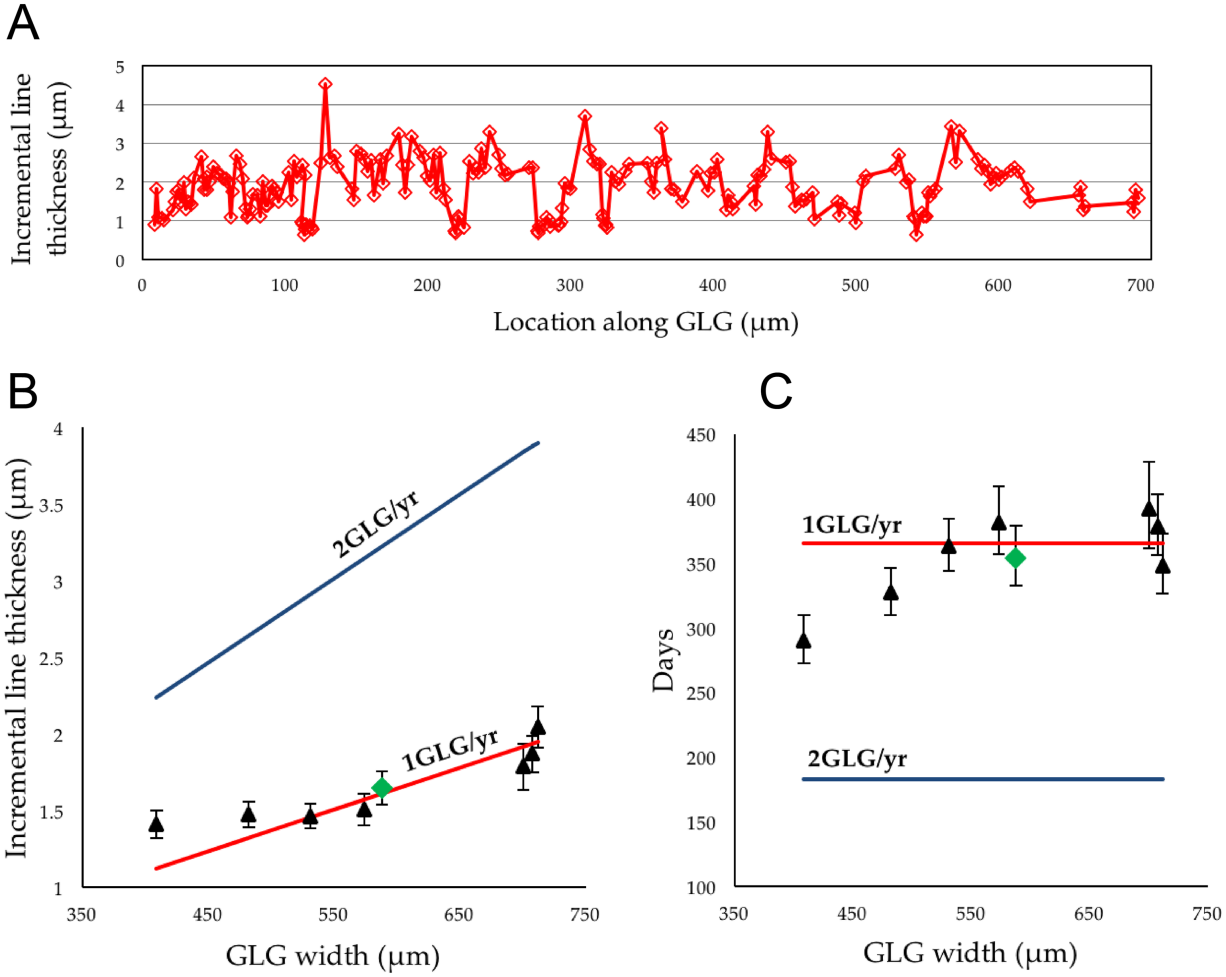
Plotted data. (A), distribution of short-period incremental lines plotted against distance from start of the GLG (2012LDL8-c); (B), mean incremental line spacing (triangles) plotted against GLG width, error bars show 3σ_SE_, solid red line represents expected spacing if GLG represents one year (1GLG/yr), solid blue line represents the expected spacing if two GLGs are deposited in a year (2GLG/yr), green diamond shows the mean of all seven GLGs; (C), estimated number of days represented in each GLG, solid lines represent expected number of days for 1GLG/yr and 2GLG/yr, green diamond shows the mean of all seven GLGs.

Seven GLGs from three whale specimens were selected for quantification of the short-period incremental line spacing. On average, almost 30% of the total thickness of the GLG contained incremental lines that were clear enough to measure. We assumed that short-period lines in difficult to read areas were the same thickness as in regions that were readable. This conservative assumption assumes that the deposition rate was constant throughout a GLG. The number of observable short-period lines per GLG varied from 113 to 220 (Table 1). To illustrate the typical density and distribution of readable incremental line spacing, individual measurements with their position along the GLG are plotted for one GLG in Fig 3A. The tabulated results for incremental line spacing from each GLG, along with GLG thickness, and estimated days represented by each GLG are presented in Tables 1 and 2, along with the predicted incremental line width for GLGs forming in both annual and semi-annual scenarios and are plotted Fig 3B and 3C. The mean incremental line spacing for all seven GLGs was 1.65 μm (3σ_SE_: 0.11), and the mean number of days estimated for the seven GLGs is 354.22 (3σ_SE_: +24.91, -21.27).

## Discussion and conclusions

### Daily incremental lines in beluga dentin

Daily incremental growth lines in dentin have been recognized in a number of taxa [45–53], and it is no surprise we observe them in beluga. Kear and von Nolting [55] may have been the first to describe them in the Odontoceti. Although the scale and periodicity or are not stated, Kiel and von Nolting described incremental lines that follow calcospheres in the dentin (discussed below) and we suggest that they observed the same type of daily incremental lines that we described in beluga. Myrick [56] may have been the first to recognize these short-period lines as representing daily incremental growth lines in the Odontoceti.

Dean [66] summarized some methods that have been used to increase the visibility of incremental lines in dentin and notes that these lines are often more apparent in fossilized dentin. For beluga teeth, we can conclude that the treatment used to extract and store the teeth did not preclude observation of incremental lines, but we cannot assess the possibility that long-term storage and maceration did not enhance incremental line visibility. Our results document that incremental lines occur in beluga dentin, and that silver or hematoxylin are suitable stains for their study.

Daily incremental lines may not always be easily observable, and their histologic expression may be variable even within different sections from a single tooth [49,53,54,66,74,77,78]. A similar situation seems to exist in beluga dentin. Although we observed short-period lines in all teeth examined, not all sections or GLGs within a section contained enough clearly resolved incremental lines to be quantified in a meaningful way. Some of this variability likely stems from the difficulty in obtaining a perfectly longitudinal section through the axis of a curved tooth.

Within a single GLG, the visibility of the short-period incremental lines seems to increase in regions containing higher densities of stained dentine tubules, so that if a series of lines are followed in a direction perpendicular to the growth of the dentin, the lines may appear to fade in and out with the apparent tubule density (Fig 2C). In this case, local histochemical reactions may be altering the expression, possibly related to differences in the amount, or nature, of the peritubular dentin which develops within the tubules [79]. Despite these difficulties, we were able to demonstrate the presence of short-period incremental lines in beluga dentin, and in numbers that allow us to test a biologically relevant hypothesis.

Dentin formation is a two-step process, in which the organic matrix is secreted and subsequently mineralized at a later time [80]. Both processes follow a circadian rhythm [80]. The advancing mineralization front is made up of discrete centers of mineralization, and in areas where these point sources do not coalesce, remnant spherical to semispherical zones known as calcospheres are produced [76,81–83]. These calcospheres, or regions of globular dentin have been previously described in the dentin of toothed whales [22,55,84]. Regions containing multiple calcospheres are known as globular dentin [80]. Fig 2A illustrates such a zone in beluga dentin. When calcospheres do coalesce, a more linear calcification front (Fig 2D) is produced [77,82]. The recognition of calcospheres, and their relationship to the short-period incremental lines in our beluga sections, (Fig 2A–2D) allow us to infer, as did Beynon et al. [73] in a fossil primate, that the short-period incremental lines we observe are the result of pulses in mineralization, and not the cyclic production of organic matrix. Given the number of studies experimentally showing that dentin mineralization proceeds in a circadian rhythm [45–53] we can conclude that the short-period incremental lines we observe in beluga represent daily increments of growth.

The relationship between the calcospheres and the incremental lines also allows us to conclude that the lines we observed in the de-mineralized sections stained with hematoxylin or silver (Fig 2A, 2C and 2D), and in the ground section (Fig 2B), are of the same origin. Decalcification and dehydration of mineralized tissues often cause the tissue to shrink [85], explaining the difference in spacing we observe in de-mineralized (1.6 μm) and ground section (2.3 μm) preparations. Dean [66] found that de-mineralized preparations in modern human teeth had incremental lines that were 25–29% closer together than in untreated ground sections, which is consistent with our observations comparing ground and demineralized preparations, although only one nondemineralized section was examined. In this context, ground sections may provide the most accurate absolute rates of dentin formation, but the periodicities remain unchanged in both ground and demineralized stained sections.

### Validation of GLG’s using daily incremental lines

Daily incremental lines have seen the widest application in the study of dentin accretion rates and the evolution of tooth formation times in modern humans, primates, and fossil hominids [75,86–88]. Daily incremental growth lines have been used to measure dentin growth and tooth replacement rates in dinosaurs [89–91]. We apply similar methodologies using daily incremental lines in beluga teeth to estimate the time represented by a single GLG.

Comparison of mean daily incremental line spacing in our samples with the predicted values for either one annual GLG or two semi-annual GLGs per year, showed clear support for the hypothesis that one GLG represents one year’s growth (Fig 3B, Table 2). As another measure, the mean number of estimated days in the seven GLGs measured is 354.22 (3σ_SE_: +24.91, -21.27), a figure that is consistent with the 365 day-long year within statistical error (Fig 2C). Our results confirm other studies that support the deposition of a single GLG per year in beluga [4,12,32–36,92], providing an independent test using a new methodology.

We have shown that measurement of daily growth increments can be used in the validation of the rate of GLG formation in beluga. This methodology may find applications in other Cetacea, especially for cases in which the logistics of marking animals with tetracycline are not feasible, and know-age animals are unavailable or where growth in captivity results in altered histological characteristics compared to those seen in wild populations. The technical hurdles are less involved than in the use of tetracycline, and issues involving the readability of GLGs in captive animals are avoided. Our methodology is complementary to existing methods, and not a replacement, although in the case of fossil or extinct taxa, validation via daily incremental lines may provide the only feasible option. That Myrick [56] was able to resolve daily lines in a dolphin sample provides optimism for potential broader applicability.
